# The Relationship among Dyadic Adjustment and Disease Burden in Patients with Bipolar Disorder and Their Spouses

**DOI:** 10.3390/bs13020091

**Published:** 2023-01-22

**Authors:** Zeynep Namlı, Lut Tamam, Mehmet Emin Demirkol, Mahmut Onur Karaytuğ, Caner Yeşiloğlu, Kerim Uğur

**Affiliations:** 1 Medical School, Department of Psychiatry, Çukurova University, 01330 Adana, Turkey; 2 Department of Psychiatry, Ahi Evran University, 40100 Kırşehir, Turkey; 3 Medical School, Department of Psychiatry, Turgut Özal University, 44900 Malatya, Turkey

**Keywords:** bipolar disorder, spouse, dyadic adjustment, burden, alexithymia, sexual functions

## Abstract

(1) Background: Spouses of individuals with bipolar disorder (BD) experience significant burdens, and the perception of the burden may affect dyadic adjustment. We aimed to investigate the sexual functions, alexithymic traits, marital satisfaction, and burden in patients with BD and their spouses. We also aimed to assess the mediating role of sexual functions and alexithymia in the relationship between burden and dyadic adjustment. (2) Methods: We included 81 patients with BD type 1 (40.69 ± 8.55 years, 65.4% female, and 34.6% male) and their healthy spouses (40.95 ± 7.30 years, 34.6% female, and 65.4% male) and 78 healthy controls (38.90 ± 5.88, 48.7% female, and 51.3% male). The participants were evaluated using the Golombok–Rust Inventory of Sexual Satisfaction (GRISS), Dyadic Adjustment Scale (DAS), Hamilton Depression Rating Scale (HDRS), Toronto Alexithymia Scale-20 (TAS-20), and Burden Assessment Scale (BAS). (3) Results: The GRISS scores of the control group were significantly lower than the spouses and BD groups. The DAS total score of the control group was significantly higher than that of the spouses and BD groups. Regression analyses revealed that TAS, GRISS, and HDRS scores were associated with DAS scores in the BD group. In the spouse group, TAS and BAS scores were associated with DAS scores. The GRISS scores partially mediated the relationship between dyadic adjustment and burden in the spouses of patients with BD. (4) Conclusions: Mental health professionals should regularly scan caregivers’ perceptions of burden. Appropriate psychosocial interventions could help spouses of patients with BD to cope better with the burden and improve dyadic adjustment.

## 1. Introduction

Bipolar disorder (BD) is a chronic psychiatric disorder with recurrent episodes and periods of well-being [1]. Chronic psychiatric disorders can cause disability in various areas and social and economic difficulties, and 10% of the patients with BD need help in their daily activities in the later stages of the disease [2,3].

Caregiver burden is the presence of difficulties and adverse events that affect the emotional, social, financial, physical, and spiritual functions of family members and friends of psychiatric patients [4]. The factors affecting the burden include the severity, nature, and duration of the disease symptoms, the status of receiving treatment, the presence of comorbid diagnosis, the sociodemographic characteristics of the patient and the caregiver, and the mental and health status of the caregiver [3,5]. Previous studies have reported deterioration in social relationships and quality of life [6], health problems, increased stress, depressive symptoms, more referrals to health institutions, and increased mortality rates in caregivers [4,7]. The caregiver burden has been primarily investigated in severe and persistent disorders such as schizophrenia and dementia [8]. The World Health Organization stated that BD causes disability at close rates to schizophrenia in young people [4,9], and patients with BD also create a care burden for their families [10,11]. Ninety-three percent of the families of patients with BD reported that they experienced a burden associated with the disease [12]. Clinical features, such as the age of onset, the predominance of manic/depressive episodes, and inter-episodic symptoms, may affect the caregiver burden [4]. Hospitalization may negatively affect relationships, career, and financial independence; manic symptoms may cause the risk of harm to the patient and their caregivers, and untreated interim periods and depressive symptoms may cause loss of functionality in patients with BD [4,12].

Whisman identified the dyadic adjustment as perceived satisfaction based on partners’ communication and interactions, although differing perspectives exist [13]. The long-term consequences of BD could lead to deterioration in marriage and relationships [14], and most patients with BD are single or divorced [15]. Changes in the family environment due to the disease, socioeconomic difficulties, disease periods, and the types of episodes affect dyadic adjustment [16]. Perlick et al. determined that spouses experience a more severe burden in their study of caregiver burden in BD [7].

Sexual satisfaction is one of the essential factors in dyadic adjustment, and there may be a bidirectional relationship between sexual dysfunctions and marital problems [17]. Sexual and marital dissatisfaction is also associated with higher burden perception in spouses of people with chronic diseases [18]. Previous studies have shown that patients with BD report more sexual problems than healthy controls [19,20]. Fewer studies evaluated the spouses of patients with BD and revealed a decrease in sexual satisfaction in spouses after the onset of the disease [16,21].

Alexithymia, defined as difficulty defining emotions, is a multifaceted concept associated with BD [22]. Previous research reported that individuals with alexithymia might show higher psychological distress than those without, and individuals unaware of their feelings had lower marital satisfaction [23]. Alexithymia was associated with increased caregiver burden and decreased quality of life in caregivers of people with chronic diseases [24].

The increase in caregivers’ burden in BD negatively affects the course of the disease [7,25]. Determining the factors associated with the disease burden in patients with BD and planning the essential interventions would positively affect dyadic adjustment and the disease course. Previous studies frequently investigated the relationship between disease severity, clinical course, sociodemographic data, and dyadic adjustment, disease burden in patients with BD [3,4,5,16]. For this rationale, we investigated the relationship between burden and dyadic adjustment with factors such as alexithymia and sexual functions, often beyond the known clinical features. We aimed to evaluate the relationship between disease burden and dyadic adjustment in patients with BD and their spouses and to examine clinical variables such as sexual functions and alexithymia that may be effective in this relationship. Our study investigated two hypotheses: (1) Dyadic adjustment and sexual satisfaction in the patients with BD and their spouses would be lower than in healthy controls; (2) Sexual functions and alexithymia would mediate the relationship between dyadic adjustment and burden in the spouses.

## 2. Method

### 2.1. Sample and Procedure

We conducted the study in the Çukurova University Faculty of Medicine Department of Psychiatry BD outpatient unit. We included only outpatients because using multiple psychotropic medications, accompanying psychotic symptoms, and symptom severity of patients with BD hospitalized for manic or depressive episodes would affect their compliance with psychiatric interviews and self-report scales. We included only literate participants to complete the self-report scales. The study sample consists of three groups: patients with BD type 1 and their healthy spouses and healthy controls without a diagnosis of mental disorders in themselves or their spouses. The inclusion criteria for the BD group were: (1) Aged 18–60 years; (2) Have been married for at least one year; (3) No diagnosis of neurocognitive disorders such as dementia or mental retardation; (4) No diagnosis of physical illness; (5) Being literate; (6) On continuous treatment with only mood stabilizers (lithium or divalproex sodium) and no hospitalization or medication change in the last three months; (7) Obtaining a score of six or less on the Young Mania Rating Scale (YMRS) and eight or less on the Hamilton Depression Rating Scale (HDRS) [26]; (8) No diagnosis of alcohol/substance use disorder. The inclusion criteria for the control group were: (1) Aged 18–60 years; (2) Have been married for at least one year; (3) Being literate; (4) No diagnosis of mental disorders; (5) Not being the spouse of a patient with a psychiatric disorder.

The first author conducted a psychiatric interview with the participants based on the Diagnostic and Statistical Manual of Mental Disorders fifth edition (DSM-5) criteria [27]. We allocated approximately 40–60 min to each participant for the psychiatric interview and filling out the forms. The interviewer explained the points that the participants did not understand.

During the psychiatric interviews, the following patients (and their spouses) were excluded: four patients who were not to be in the euthymic period, five patients diagnosed with comorbid alcohol-substance use disorder, five with generalized anxiety disorder, and seven patients whose spouses were diagnosed with major depressive disorder. We excluded four participants from the control group with anxiety disorder and three who did not complete the forms. Consequently, we conducted the study with 81 BD patients and their spouses and 78 healthy controls.

Before the study, all participants filled out an informed consent form. Çukurova University Faculty of Medicine Non-Invasive Clinical Research Ethics Committee approved the study (meeting no. 95, dated 10 January 2020, decision no. 29).

### 2.2. Measures

#### 2.2.1. Sociodemographic and Clinical Data Form

The data form evaluated the participants’ sociodemographic characteristics, such as age, gender, educational status, employment status, and duration of the marriage, and disease characteristics, such as disease duration, number and types of episodes, hospitalization, and medications.

#### 2.2.2. The Young Mania Rating Scale (YMRS)

The YMRS rates the severity of mania and consists of 11 items; each item measures five-stage symptom severity. The clinician scores the scale based on the patient’s reports and observations during the interview [28]. In the Turkish validity and reliability study, Cronbach’s alpha value was 0.79 [29], which we found as 0.75. 

#### 2.2.3. The Hamilton Depression Rating Scale (HDRS)

The HDRS measures the severity of depression and is administered by the interviewer. It consists of 17 items, and the maximum score from the scale is 51. High scores indicate increased severity of depressive symptoms [30]. In the Turkish validity and reliability study, Cronbach’s alpha value was 0.75 [31], and we found as 0.81.

#### 2.2.4. The Golombok–Rust Inventory of Sexual Satisfaction (GRISS)

The GRISS determines the nature of sexual intercourse and evaluates sexual dysfunctions. It is a self-report scale consisting of 28 items for men and women, and raw scores are converted into standard scores ranging from one to nine. High scores indicate deterioration in sexual functions and the quality of the relationship [32]. In the Turkish validity and reliability study, Cronbach’s alpha value was 0.92 for men and 0.91 for women [33], which we found as 0.87 for men and 0.84 for women.

#### 2.2.5. The Dyadic Adjustment Scale (DAS)

The DAS is a 32-item, multidimensional self-report scale [34]. It consists of four subscales: dyadic satisfaction, dyadic cohesion, dyadic consensus, and affectional expression. The score range is 0–151, and a higher total score indicates a better relationship or marital adjustment. In the Turkish validity and reliability study, Cronbach’s alpha value was 0.92 [35], and we found it as 0.88.

#### 2.2.6. The Toronto Alexithymia Scale-20 (TAS-20)

The TAS is a five-point Likert-type self-report scale comprising 20 items, and high scores indicate higher alexithymic symptoms [36]. In the Turkish validity and reliability study, Cronbach’s alpha value was 0.78 [37], which we found as 0.83.

#### 2.2.7. The Burden Assessment Scale (BAS)

The BAS determines the extent to which the daily lives of caregivers of psychiatric patients are affected by the disease and the extent to which they are concerned about their relatives, regardless of symptom severity. It is a 19-item four-point Likert-type self-report scale and assesses the burden of families with a seriously mentally ill member, such as mood disorders [38]. In the Turkish validity and reliability study, Cronbach’s alpha value was 0.89, and we found it as 0.81. As the total scale score increases, the perception of disease burden also increases [10].

## 3. Statistical Analysis

IBM SPSS 25 program was used in the analysis of the data. In cases where the skewness and kurtosis values were between −1.5 and +1.5, the z-score was between −3.29 and 3.29; evaluating the histograms, the variable distributions were considered to be normally distributed. The means of normally distributed variables were compared with ANOVA and Brown–Forsythe, and were shown as mean and standard deviations (mean ± SD). The medians of the non-normally distributed variables were compared with Kruskal–Wallis and shown as medians and quartiles (1st quarter–3rd quarter).

The relationship between the categorical variables was analyzed using Fisher Exact (or Fisher–Freeman–Halton for RxC tables) if the expected number of observations was less than 5, Yates’ statistic if it was between 5 and 25, and the Chi-square test in other cases and shown in tables with frequencies and percentages.

The Bonferroni correction was applied for multiple comparisons, and differences were detected. Pearson correlation analysis was used to determine the relationship between the scales. Multiple linear regression analysis was applied separately for the BD and spouse groups by choosing the DAS as the dependent variable. All assumptions necessary for applying the multiple linear regression model were provided.

Only in the spouse group two different models were formed in which the TAS and GRISS scales were mediating variables. In the first model, the mediating role of sexual functions in the relationship between dyadic adjustment and disease burden was investigated. The second model examined the mediating role of alexithymia in the relationship between dyadic adjustment and the burden. The mediation analyses were tested with the SPSS Process Macro plugin model 4, with 5000 bootstrapped samples [39]. 

The significance level was accepted as 0.05 for all analyses, and statistically significant values were bold in the tables.

## 4. Results

Table 1 presents the sociodemographic characteristics of the sample. The groups were similar in terms of age, educational status, marriage duration, and place of residence (Table 1, *p* > 0.05, for each parameter). There was a significant relationship between gender and groups (*p* < 0.001). According to the post hoc test with Bonferroni correction, the proportion of women in the BD group was higher than in the spouse group with a weak effect size. There was a significant relationship between occupational status and groups (*p* < 0.001). According to the post hoc test with Bonferroni correction, the rates in all groups differ. While the proportion of non-working individuals was higher in the BD group than in all groups, the highest proportion of working individuals was in the control group. The effect size between the groups was moderate. There was a significant relationship between the family history of mental disorders and the groups (*p* < 0.001). With the post hoc test, the family history of mental disorders was higher in the BD group than in the spouse and control groups, and the effect size between the groups was moderate.

In patients with BD, the average duration of the disease was 12 years, and the average number of hospitalizations was two. Twenty-nine (35.8%) of the patients attempted suicide at least once in their lifetime. The median number of manic episodes was two, depressive episodes was three, and hypomanic episodes was one.

Table 2 presents the participants’ scale scores. There was a significant difference between the groups according to the mean GRISS scores (*p* < 0.001). According to the post hoc test, the mean of the control group’s GRISS scores was lower than that of the spouse and BD groups. The effect size of the difference was large (ƞ^2^ = 0.32), and the groups explained 32% of the total variance. 

There was a significant difference between the groups according to the mean DAS scores (*p* = 0.003). According to the post hoc test, the mean of the control group’s DAS scores was higher than those of the spouse and BD groups. The effect size of the difference was small (ƞ^2^ = 0.05), and the groups explained 5% of the total variance.

There was a significant difference between the groups according to the mean scores of the DAS-affectional expression subscale (*p* = 0.03). According to the post hoc test, the control group’s DAS-affectional expression mean scores were higher than the BD group. The effect size of the difference was small (ƞ^2^ = 0.03), and the groups explained 3% of the total variance.

There was a significant difference between the groups according to the mean scores of the DAS-dyadic cohesion subscale (*p* = 0.02). According to the post hoc test, the control group’s DAS-dyadic cohesion mean scores were higher than those of the BD and spouse groups. The effect size of the difference was small (ƞ^2^ = 0.03), and the groups explained 3% of the total variance.

There was a significant difference between the groups according to the median of the DAS-dyadic satisfaction subscale scores (*p* < 0.001). In pairwise comparisons, the control group’s DAS-dyadic satisfaction scores were higher than those of the BD and spouse groups. The effect size of the difference was medium (ƞ^2^ = 0.10), and the groups explained 10% of the total variance.

There was no significant difference between the groups in terms of the mean scores of the TAS and DAS-dyadic consensus subscale (*p* = 0.051; *p* = 0.21, respectively).

There was no statistically significant relationship between DAS scores and age, education period, marriage, and disease duration in patients with BD (*p* > 0.05, for each parameter). A statistically significant, moderate, and inverse linear relationship existed between DAS and TAS and between DAS and GRISS scores (*p* < 0.001 for each) (Table 3). 

There was no statistically significant relationship between DAS scores and age, education period, and marriage duration in the spouses’ group (*p* > 0.05, for each parameter). There was a statistically significant, weak, and inverse relationship between DAS scores and TAS and GRISS scores (*p* = 0.002; *p* = 0.003, respectively) (Table 3).

There was no statistically significant relationship between BAS scores and age, education period, and marriage duration in the spouses’ group (*p* > 0.05, for each parameter). A statistically significant, moderate, and inverse linear relationship existed in the spouses’ group between DAS and BAS scores (*p* < 0.001). There was a statistically significant, weak, and same-direction relationship between BAS and TAS scores (*p* = 0.002) and a moderate, same-direction relationship between BAS and GRISS scores (*p* < 0.001) (Table 3).

A multiple linear regression model was designed by containing the variables found to be related in the correlation analysis with the DAS and the variables of gender and occupational status only in individuals with BD. The independent variables explained 31% of the DAS. The TAS predicted the DAS negatively. A one-unit change in the TAS induced a 0.68-unit decrease in the DAS. The GRISS predicted the DAS negatively. A one-unit change in the GRISS induced a 4.32-unit decrease in the DAS. The HDRS predicted DAS negatively. A one-unit change in the HDRS caused a 2.74-unit decrease in the DAS. Gender and occupational status did not statistically predict the DAS (Table 4).

A multiple linear regression model was designed by adding the variables found to be related in the correlation analysis with the DAS, and the variables of gender and occupational status, only in the spouses’ group. The independent variables explained 31% of the DAS. The TAS predicted the DAS negatively. A one-unit change in the TAS led to a 0.50-unit decrease in the DAS. The BAS predicted the DAS negatively. A one-unit change in the BAS caused a 0.80-unit decrease in the DAS. The GRISS, the HDRS, gender, and occupational status did not statistically predict the DAS (Table 4).

### Mediation Analyses

*Subproblem 1:* What is the mediating role of sexual functions in the relationship between dyadic adjustment and burden in spouses of patients with BD?

The following analysis was performed to test the hypothesis. In this analysis, the response variable (Y) was the burden (BAS score), the independent variable (X) was the dyadic adjustment (DAS score), and the mediating variable (M) was the sexual functions (GRISS score).

**Figure 1:**
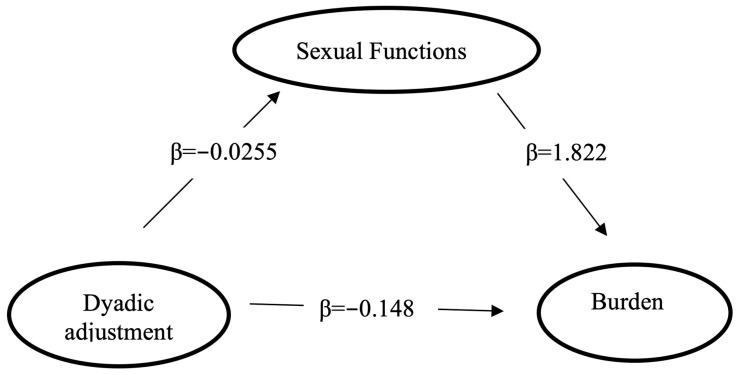
The mediating role of sexual functions in the relationship between dyadic adjusment and burden in the spouses.

Dyadic adjustment significantly and negatively predicted disease burden without adding the mediator variable (GRISS score) (β = −0.1948; *p* < 0.001). After adding the mediator variable (GRISS) to the model, dyadic adjustment predicted the mediator (β = −0.0255; *p* = 0.003), and the mediator predicted the disease burden (β = 1.8226; *p* = 0.0005). Figure 1 presents the path diagram containing the standardized path coefficients of the mediation analyses to which the GRISS score was added. According to the data, sexual functions partially mediated the relationship between dyadic adjustment and disease burden (Table 5, Figure 1).

*Subproblem 2:* What is the mediating role of alexithymia in the relationship between dyadic adjustment and burden in spouses of patients with BD?

The following analysis was performed to test the hypothesis. In this analysis, the response variable (Y) was the burden (BAS score), the independent variable (X) was the dyadic adjustment (DAS score), and the mediating variable (M) was the alexithymia (TAS score).

Dyadic adjustment significantly and negatively predicted disease burden without adding the mediator variable (TAS score) (β = −0.1948; *p* < 0.001). After adding the mediator variable (TAS) to the model, dyadic adjustment predicted the mediator (β = −0.1470; *p* = 0.002), and the mediator predicted the disease burden (β = 0.1879; *p* = 0.0621). According to bootstrap confidence intervals, alexithymia did not mediate the relationship between dyadic adjustment and disease burden (Table 6). Therefore, the mediation analysis was not modeled with a diagram.

## 5. Discussion 

BD is a chronic mental disorder that induces significant consequences, such as disability and loss of functionality, in the patient and causes a considerable care burden for caregivers. Caregivers of patients with BD report severe burden perception, anxiety, depression, and distress [40,41]. High care burden affects the patient, caregiver, and the clinical course of the disease in a vicious circle [41]. Spouses of individuals with mental disorders also perceive a burden, and the perception of a high burden negatively affects marital adjustment [42]. Previous research has suggested identifying the effects of mediators on the relationship between BD patients and their caregivers [43]. The most remarkable result of our study was that patients with BD and their healthy spouses had a lower dyadic adjustment and sexual satisfaction than healthy controls, and there was a negative correlation between alexithymic characteristics, sexual dissatisfaction, and dyadic adjustment in both groups. Another significant finding of our study was that sexual functions partially mediated the relationship between dyadic adjustment and burden in the spouses’ group.

Previous research has reported that a BD diagnosis in one spouse is an essential indicator of marital conflict and has noted high levels of dissatisfaction [44]. However, there is conflicting evidence on this point, and some studies note little marital conflict in patients with BD compared to healthy couples [16,45]. Although studies on marriage and dyadic adjustment in BD are affected due to cultural factors, they have shown that difficulties are experienced primarily in communication areas [16]. In our study, patients with BD and their spouses reported similar levels of dyadic adjustment. In contrast, healthy couples’ adjustment level was significantly higher with a small effect size, and patients with BD and their spouses were affected in all areas of adjustment except dyadic consensus. Conflicting findings may be related to the need for more use of standardized tools in a significant portion of the literature [16]. In addition, the lack of evaluation for different periods, as in our study, may affect the spouses’ perception of dyadic adjustment. For example, while spouses may interact less with patients during manic episodes, they may share more sadness in depressive episodes.

Patients with BD have reported deterioration in their sexual functions. Dargel et al. revealed that patients with BD in remission have significantly more sexual dysfunctions than healthy individuals [46]. Sexual dysfunction has been more common in spouses of patients with BD than in healthy individuals [47]. Similarly, in our study, patients with BD in remission and their spouses reported similar sexual satisfaction. In contrast, the sexual satisfaction of healthy individuals was significantly higher with a large effect size. Marital satisfaction has been associated with sexual functioning. In the course of the BD, spouses may feel more anxious during manic episodes, may have difficulty coping with the increase in the patient’s sexual urges, and thus, may avoid close contact. In contrast, during depressive episodes, spouses may have difficulty managing the patient’s loss of libido [16]. Previous studies have shown that sexual dissatisfaction was associated with marital satisfaction in spouses of patients with BD [47,48]. In our study, the decrease in dyadic adjustment as both the patients with BD and their spouses increased sexual dissatisfaction confirms previous studies. However, our study could not determine the direction of causality between these two variables. Therefore, it is possible that sexual problems lead to dyadic problems or that dyadic problems lead to sexual problems. Longitudinal studies are needed to investigate how these variables are related over time.

Deterioration in psychosocial functionality has been observed in patients with BD, including remission periods [49]. Research has shown the limited predictive ability of clinical and demographic variables, such as age and number of hospitalizations, in assessing functionality [50], and these data suggest that additional factors significantly affect functionality. Social cognitive deficits, especially emotion recognition and theory of mind, have been observed in patients with BD [51,52]. Although some dimensions of social cognition, such as the theory of mind and emotion recognition, have been widely investigated, alexithymia remains largely unexplored. Ospina et al. demonstrated that alexithymic domains were predictors of social functioning in patients with BD [22]. In our study, the negative correlation between alexithymic traits and dyadic satisfaction in patients with BD and their spouses could be explained by the fact that individuals with alexithymia avoid talking about their feelings, have impaired emotional functioning, and have difficulties in interpersonal relationships [22]. Consistent with our results, previous research reported that alexithymia predicted socio-emotional functioning in non-clinical populations and psychiatric disorders [53]. Alexithymia is also characterized by an inability to distinguish between emotions and bodily sensations and a limitation in fantasy life [54]. Regression analyses indicated that alexithymia was essential in patients with BD and their spouses’ perceptions of dyadic adjustment. Our results suggest the importance of therapeutic interventions to improve emotional awareness to increase social functioning and dyadic adjustment in patients with BD and their spouses. However, our result that the groups did not differ in terms of alexithymic characteristics suggests that alexithymia is not specific to BD and could be characterized as a personality trait. A theoretical perspective divides alexithymia into the absence of emotional experience and a selective lack of emotional cognition in which emotional experience is preserved [55]. Developing an assessment of alexithymia for subtypes [22] could help differentiate the clinical and non-clinical populations.

Patients with BD with subsyndromal depressive symptoms are likely to report impaired family and friendship relationships and impaired work and home functioning roles [56]. Sheets and Miller showed that the depressive symptomatology of patients affected their perceptions of general family functioning and couple functioning [57]. The regression analysis results in our study revealed that depressive symptoms were associated with the perception of dyadic adjustment of patients with BD. In line with previous studies, our results support that subthreshold depressive symptoms affect the social functioning of patients with BD in the euthymic period.

Previous research assessed differences in caregiver burden in various populations globally. Some studies have reported that caregivers of patients with psychiatric disorders feel more burden than those with other chronic medical illnesses [58,59]. However, the perception of burden is highly subjective and variable due to cultural factors [60]. Tanna has shown that primary caregivers of patients with BD feel a moderate-to-severe burden, according to the Zarit Burden Interview scores [60]. 

The perception of burden differs according to the caregiver’s relationship with the patient. While caregiving in patients with schizophrenia is often the responsibility of the patient’s parents, this role is assumed mainly by spouses in BD [61,62]. However, spouses face disease-specific burdens and burdens arising from partnership and family roles. These multiple burdens lead to high levels of marital distress, with many spouses reporting significant distress and reduced couple adjustment, often leading to separation [63,64]. Similarly, in our study, dyadic adjustment decreased as the burden perception of spouses increased. The relationship between dyadic adjustment and burden perception could be interpreted bidirectionally; that is, as spouses’ satisfaction with the relationship increases, burden perception may decrease.

Caregiver burden is a multifaceted concept associated with several variables other than patient-related factors, such as changes in caregiver functioning, relationship functioning, psychiatric symptoms, and caregiver coping skills [43]. Spouses of patients with BD noted that sexual relationship problems persisted even when the patients were in remission [65]. Previous studies have indicated that spouses perceived more burden due to experiencing sexual dissatisfaction [66]. We found that as the alexithymic traits and sexual dissatisfaction of the spouses increased, the perception of the burden increased. According to the regression analysis results, one of the most critical variables for spouses’ relationship satisfaction was the perceived disease burden. To reduce the high divorce rates in patients with BD [67], mental health professionals should provide individual and couple psychosocial support to both patients and their spouses.

Caregiver burden leads to the caregiver’s poor mental and physical well-being and reduced self-care and social support. Over time, the burden increases the frequency with which caregivers seek healthcare services, and the high burden leads to reduced caregiving effectiveness. The perceived high burden of caregivers negatively affects patients’ compliance and disease course [64]. For these reasons, reducing caregivers’ burden and increasing their coping skills should be a mental health priority. In our study, in mediation analyses, only sexual functions, not alexithymic characteristics, had a partial mediator role in the relationship between spouses’ relationship satisfaction and burden perception. Previous research has pointed out that family-oriented cognitive behavioral therapy and psychoeducational programs lead to positive changes in caregivers, and these changes result in a decrease in patients’ symptoms [68,69]. According to our results, evaluating from the perspective of the spouse and planning interventions, especially for sexual functions, will reduce the perception of disease burden, increase dyadic adjustment, and may positively affect the course of the disease.

BD is one of the most heritable psychiatric disorders, and the incidence of BD risk in first-degree relatives of patients with BD is estimated to be approximately ten times that of the general population [70]. Moreover, despite optimal pharmacotherapy, social and cognitive dysfunctions remain a significant problem in bipolar disorder. Some studies have suggested that social and cognitive dysfunction persists in patients with BD not only during the acute phase of the illness but also during remission. In 60–70% of patients with BD, occupational functioning is reported to be impaired to varying degrees [71]. In the present study, the high rates of unemployment and family history of mental disorders in patients with BD suggest that our sample is representative of patients diagnosed with BD.

## 6. Limitations and Future Directions

Our study includes some limitations. First, the cross-sectional design prevents the establishment of a cause-and-effect relationship. Second, the predominance of married female patients in our BD outpatient clinic resulted in an unbalanced gender distribution in the patient group. The predominance of female patients and the evaluation of patients followed in a single university hospital may not reflect cultural differences and may prevent the generalization of the results. The third limitation is that drug treatments were not evaluated as they may affect sexual functioning. Including only literate individuals may limit results, and using multiple questionnaires may have made it difficult for patients with BD to adapt. In addition, the evaluation of currently cohabitating spouses does not reflect the caregiver burden of divorced or separated partners. In a large sample with a balanced gender distribution, follow-up studies, including the periods of episodes, will enlighten the subject and guide the planning of interventions to increase dyadic adjustment in BD.

## 7. Conclusions

According to our results, sexual satisfaction and dyadic adjustment were lower in patients with BD and their spouses compared to healthy individuals. The essential variables in dyadic adjustment from the perspective of individuals with BD were sexual functioning, alexithymia, and subsyndromal depressive symptoms, whereas alexithymia and burden perception for spouses. In addition, another significant result of our study revealed the partial mediator role of sexual functioning in the relationship between dyadic adjustment and burden perception in spouses. Caregivers play an essential role in helping individuals with chronic mental disorders. Supporting caregivers by reducing their burden and improving their psychological functioning could help them continue to provide support. For these reasons, mental health professionals should examine the social functioning of spouses in the follow-up of patients with BD, in addition to focusing on the clinical symptoms of the disease. Interventions adapted to the psychiatric needs of the spouses of patients with BD, such as couple therapy, sexual therapy, and psychoeducation, could help to improve the mental health of the caregiver and the patient and increase dyadic adjustment.

## Figures and Tables

**Table 1:** Sociodemographic features of the participants.

	BD (n = 81)	Spouse (n = 81)	Control (n = 78)	Effect Size	*p*
Age, years	40.69 ± 8.55	40.95 ± 7.30	38.90 ± 5.88		0.16 †
Education period, years	9.99 ± 3.63	9.56 ± 4.09	10.49 ± 3.60		0.30 †
Marriage duration, years	15.21 ± 9.25	15.21 ± 9.25	12.42 ± 8.03		0.07 †
Gender				0.25	<0.001 *
Female	53 (65.4%) a	28 (34.6%) b	38 (48.7%) ab		
Male	28 (34.6%) a	53 (65.4%) b	40 (51.3%) ab		
Occupational status				0.39	<0.001 *
Unemployed Employed	52 (64.2%) a 29 (35.8%) a	30 (37%) b 51 (63%) b	14 (17.9%) c 64 (82.1%) c		
Place of residence					0.18 *
Rural	35 (43.2%)	35 (43.2%)	24 (30.8%)		
Urban	46 (56.8%)	46 (56.8%)	54 (69.2%)		
Family history of mental disorders				0.31	<0.001 *
No	47 (58%) a	64 (79%) b	70 (89.7%) b		
Yes	34 (42%) a	17 (21%) b	8 (10.3%) b		

†: Anova, Brown–Forsythe, *. Pearson Chi-Square, Fisher’s Exact or Fisher–Freeman–Halton test. BD: bipolar disorder.

**Table 2:** Comparison of the scale scores of the groups.

	BD (n = 81)	Spouse (n = 81)	Control (n = 78)	Effect Size	*p*
HDRS	3 (0–5)	0 (0–0)	1 (0–3)	0.15	*p* < 0.001 ^§^
GRISS	6.53 ± 2.26	6.04 ± 2.13	3.35 ± 1.69	0.32	*p* < 0.001 †
TAS	52.81 ± 12.08	50.25 ± 11.79	48.35 ± 10.58		0.051 †
BAS	---	44.04 ± 11.26	---	---	---
DAS	103.38 ± 27.05	105.81 ± 27.10	116.04 ± 17.36	0.05	0.003 †
Dyadic consensus	48.78 ± 12.93	47.64 ± 15.57	51.12 ± 8.22		0.21 †
Dyadic satisfaction	32 (25–39.5)	36 (29.5–41)	39.5(36.75–43)	0.1	*p* < 0.001 ^§^
Affectional expression	8.67 ± 2.95	9.30 ± 2.42	9.71 ± 1.98	0.03	0.03 †
Dyadic cohesion	14.25 ± 6.25	14.58 ± 5.23	16.56 ± 4.55	0.03	0.02 †

^§^ Kruskal–Wallis, †: Anova, Brown–Forsythe, BD: bipolar disorder, HDRS: Hamilton Depression Rating Scale, GRISS: Golombok–Rust Inventory of Sexual Satisfaction, TAS: Toronto Alexithymia Scale, BAS: Burden Assessment Scale, DAS: Dyadic Adjustment Scale.

**Table 3:** The correlations between scale scores in the BD and spouses groups.

Patients with Bipolar Disorder								
		DAS	Age	Education Period	Marriage Duration	Disease Duration	TAS	GRISS
DAS	r	1	0.14	0.12	−0.03	0.06	−0.49	−0.47
	*p*		0.22	0.29	0.80	0.60	0.00	0.00
Spouses								
DAS	r	1	0,06	0.21	−0.14		−0.34	−0.33
	*p*		0.58	0.07	0.22		0.002	0.003
BAS	r	−0.47	0.03	−0.20	0.15		0.33	0.46
	*p*	0.00	0.78	0.07	0.18		0.002	0.00

Pearson correlation, GRISS: Golombok–Rust Inventory of Sexual Satisfaction, TAS: Toronto Alexithymia Scale, BAS: Burden Assessment Scale, DAS: Dyadic Adjustment Scale.

**Table 4:** Regression models for factors related to dyadic adjustment.

**Patients with Bipolar Disorder**	**Beta [95% CI]**	**Adj. Beta [95% CI]**	***p*-Value**
TAS	−0.68 [−1.12; −0.24]	−0.30 [−0.50; −0.11]	0.003
GRISS	−4.32 [−6.65; −1.99]	−0.36 [−0.56; −0.17]	p < 0.001
Female	−7.72 [−20.39; 4.94]	−0.14 [−0.36; 0.09]	0.23
Unemployed	5.48 [−7.57; 18.53]	0.10 [−0.14; 0.33]	0.41
HDRS	−2.74 [−4.66; −0.83]	−0.27 [−0.46; −0.08]	0.01
R = 0.64 R-Square = 0.41 Adj. R-Square = 0.37			
**Spouses**	**Beta [95% CI]**	**Adj. Beta [95% CI]**	***p*-Value**
TAS	−0.50 [−0.97; −0.02]	−0.22 [−0.42; −0.01]	0.04
GRISS	−1.64 [−4.48; 1.20]	−0.13 [−0.35; 0.09]	0.25
Female	−3.96 [−15.51; 7.59]	−0.07 [−0.27; 0.13]	0.50
Unemployed	10.86 [−0.70; 22.42]	0.20 [−0.01; 0.40]	0.07
HDRS	−1.48 [−4.99; 2.03]	−0.09 [−0.30; 0.12]	0.40
BAS	−0.80 [−1.36; −0.24]	−0.33 [−0.56; −0.10]	0.01
R = 0.55 R-Square = 0.31 Adj. R-Square = 0.25			

Dependent variable: dyadic adjustment, CI: confidence interval, Adj. Adjusted, HDRS: Hamilton Depression Rating Scale, GRISS: Golombok–Rust Inventory of Sexual Satisfaction, TAS: Toronto Alexithymia Scale, BAS: Burden Assessment Scale.

**Table 5:** The mediating role of sexual functions in the relationship between dyadic adjustment and burden in the spouses.

Model	Pathway	Coefficient (β)	Standard Error	*p*
l. Basic model (without M)	X → Y	−0.1948	0.0413	0.0000
ll. Mediation analysis	X → M	−0.0255	0.084	0.0031
ll. Mediation analysis	M → Y	1.8226	0.5196	0.0005
ll. Mediation analysis (direct effect)	X → Y	−0.1483	0.0408	0.0008
ll. Mediation analysis (indirect effect)	Pathway	Coefficient (β)	Standard Error	Bootstrap confidence interval BootLower limit/BootUpper limit
	X → M → Y	−0.0465	0.0176	−0.0843/−0.0151
R-Square = 0.33 Adjusted R-Square = 0.31				

**Table 6:** The mediating role of alexithymia in the relationship between dyadic adjustment and burden in the spouses.

Model	Pathway	Coefficient (β)	Standard Error	p
l. Basic model (without M)	X → Y	−0.1948	0.0413	0.0000
ll. Mediation analysis	X → M	−0.1470	0.0460	0.0020
ll. Mediation analysis	M → Y	0.1879	0.0993	0.0621
ll. Mediation analysis (direct effect)	X → Y	−0.1672	0.0432	0.0002
ll. Mediation analysis (indirect effect)	Pathway	Coefficient (β)	Standard Error	Bootstrap confidence interval BootLower limit/BootUpper limit
	X → M→ Y	−0.0276	0.0173	−0.0655/0.0014
	R-Square = 0.25 Adjusted R-Square = 0.24			

## Data Availability

The data presented in this study are available on request from the corresponding author.
